# Membrane transporters and drought resistance – a complex issue

**DOI:** 10.3389/fpls.2014.00687

**Published:** 2014-12-04

**Authors:** Karolina M. Jarzyniak, Michał Jasiński

**Affiliations:** ^1^Laboratory of Plant Molecular Physiology, Department of Natural Products Biochemistry, Institute of Bioorganic Chemistry Polish Academy of SciencesPoznań, Poland; ^2^Laboratory of Molecular Biology, Department of Biochemistry and Biotechnology, University of Life SciencesPoznań, Poland

**Keywords:** drought avoidance, drought tolerance, transmembrane allocation, transport systems, abscisic acid

## Abstract

Land plants have evolved complex adaptation strategies to survive changes in water status in the environment. Understanding the molecular nature of such adaptive changes allows the development of rapid innovations to improve crop performance. Plant membrane transport systems play a significant role when adjusting to water scarcity. Here we put proteins participating in transmembrane allocations of various molecules in the context of stomatal, cuticular, and root responses, representing a part of the drought resistance strategy. Their role in the transport of signaling molecules, ions or osmolytes is summarized and the challenge of the forthcoming research, resulting from the recent discoveries, is highlighted.

## INTRODUCTION

Drought is one of the most acute abiotic stressors, commonly encountered by plants; that adversely affects crop production worldwide. Today, the development of plants with an increased capacity to survive during water scarcity combined with a high yield potential is the principal objective of agrobiotechnology.

Over the course of evolution, plants have developed sophisticated mechanisms that allow adaptation and survival during periods of water deficit. These mechanisms serve as candidate pathways for the engineering of enhanced drought stress tolerance. In response to water deprivation, plants exhibit the following strategies: (i) escape, (ii) avoidance, and (iii) tolerance. Drought-escaping plants, such as desert ephemerals, annual crops and pasture plants, complete their life cycle before the onset of acute drought. This is achieved through high metabolic activity, rapid growth, and the acceleration of flowering. Avoidance responses occur in both annual and perennial plants. Such strategies rely on the maintenance of water uptake through the modulation of root architecture. Water loss is also limited by reducing stomatal and cuticular conductance and evaporative surfaces. Drought tolerance is associated with the ability of tissues to withstand low water potential; this is achieved via osmotic adjustment and the synthesis of low-molecular weight proteins that protect plants from damage caused by water deficiency (Bacelar et al., 2012).

Recent progress in plant functional genomics has enabled the identification and characterization of genes involved in many essential steps of the drought stress response. Increasing evidence indicates that various plant membrane transport systems play a significant role in adaptation to drought. Depending on energy needs, translocation through biological membranes occurs in a passive or active manner. Passive transport, also known as facilitated diffusion, occurs through channels and carriers. Active transport is performed by primary and secondary transporters that use ATP hydrolysis and ion gradients, respectively, to drive solutes across membranes (Schulz, 2011). The study of transporters related to signaling molecules, e.g., abscisic acid (ABA), as well as ions and osmolytes paves the way for the development of new drought-tolerant varieties. In this review, membrane transport systems will be presented according to their participation in drought avoidance and tolerance strategies. The role of various transport systems in ABA translocation, stomatal, cuticular, and root responses, as well as osmotic adjustment will be summarized.

## ABA IN DROUGHT AVOIDANCE AND TOLERANCE STRATEGIES

Abscisic acid is a ubiquitous plant hormone and signaling molecule; it controls plant growth and development and modulates the response to environmental stressors. As a phytohormone crucial in drought avoidance strategies, ABA elicits two distinct responses: rapid and gradual. The earliest plant reaction, regulated in an ABA-dependent manner, is the modulation of stomatal aperture; this minimizes the loss of water through transpiration. Exposure to ABA prompts guard cells to decrease their volume and close across the airway pore. This is achieved via changes in ion flux within the guard cell (Finkelstein, 2013). ABA gradually increases hydraulic conductivity and promotes cell elongation in the root, enabling the plant to recover after water shortage (Kudoyarova et al., 2011). By inducing the accumulation of osmotically active compounds, which protects cells from damage, ABA participates in the drought tolerance response (Finkelstein, 2013).

Biosynthetic pathways that result in free, active ABA have been previously reviewed (Finkelstein, 2013). ABA is synthesized from precursors, mainly carotenoids and xanthophylls found in plastids. The accumulation of synthesis enzymes has been observed in the veins of vegetative tissues, guard cells, maternal tissues, and embryos (Boursiac et al., 2013). Free ABA is also generated by the deconjugation of the ABA glucosyl ester (ABA-GE), a major ABA conjugate that functions as an ABA reservoir in the vacuole and endoplasmic reticulum (ER; Lee et al., 2006; Xu et al., 2012; Burla et al., 2013). According to the recent findings, the location of ABA biosynthesis depends on the water status in the environment. In the early stages of soil water deficiency, a root-derived SO_4_^2-^ signal is transported to the shoots. Consequently, ABA is synthesized in the veins of vegetative tissues and promotes stomatal closure (Ernst et al., 2010). ABA biosynthesis also occurs directly in the guard cells in response to low humidity; this ABA is assumed to be sufficient for stomata closure (Bauer et al., 2013). Continuing water shortage results in ABA biosynthesis in the roots and the redistribution of root-derived ABA through the xylem into the aerial parts of the plant (Goodger and Schachtman, 2010).

### ABA TRANSPORTERS

Recently, the molecular basis of ABA transport has been defined. Two models of transmembrane ABA translocation, based on diffusion and the presence of primary and secondary transporters, are now widely accepted. ABA, as a weak acid, exists in an anionic (ABA^-^), and in a protonated (ABAH) form. The latter, when uncharged, is able to diffuse through the plasma membrane (PM). The diffusion of weak acids has its limitations. An increase in pH (ABA pKa 4.7) due to drought stress can drastically decrease the pool of apoplastic-diffusible ABAH. This limiting step highlights the necessity of ABA transporters, which increase the amount of ABA accessible to intracellular receptors upon stress conditions (Boursiac et al., 2013).

#### Primary ABA transporters

**FIGURE 1:**
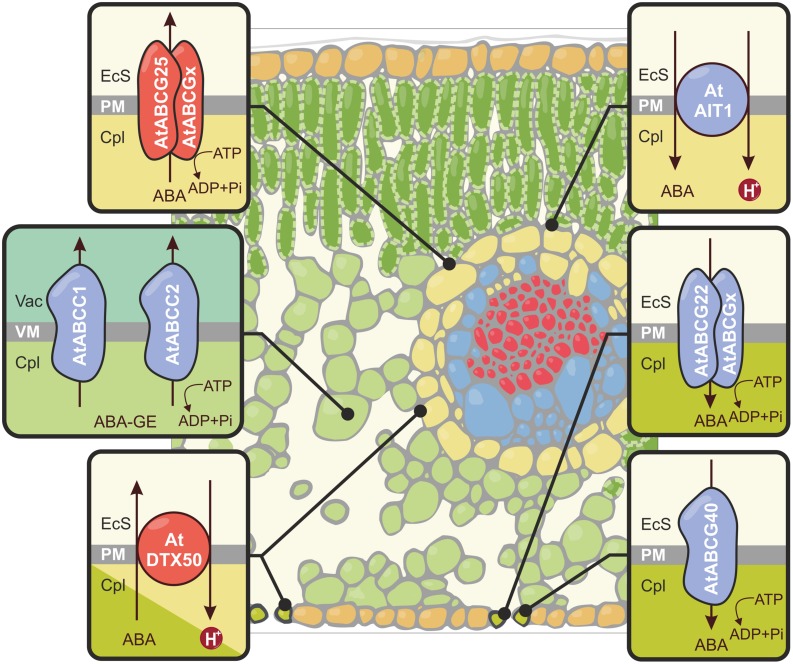
**Schematic representation of abscisic acid (ABA) and ABA glucosyl ester (ABA-GE) translocation across the plasma membrane (PM) and the tonoplast (VM) in leaf tissue.** The transporters that function as exporters (AtABCG25, AtDTX50) are shown in red, and importers (AtABCG40, AtABCG22, AtAIT1/NRT1.2, AtABCC1, AtABCC2) are presented in blue. To fulfill their role, the half-size ABC transporters form homo- or heterodimers. The composition of AtABCG25 and AtABCG22 dimers has not been yet determined. EcS, extracellular space; Cpl, cytoplasm; Vac, vacuole.

In *Arabidopsis thaliana*, members of ATP-binding cassette (ABC) family have been shown to be involved in ABA transport. The ABC proteins have been grouped into eight major subfamilies (A–H); these subfamilies correspond to phylogenetic pathways and structural features. In regard to protein architecture, the largest known subfamily of ABC proteins is the G subfamily. The G subfamily (ABCG) has been divided into the following two distinct groups: (i) the half-size transporters, which were formerly known as the white brown complex (WBC), and (ii) the full-size transporters (also known as the pleiotropic drug resistance transporters – PDRs). ABCGs comprise a single or double set of two basic structural elements: a transmembrane domain (TMD) and a nucleotide-binding domain (NBD) (Verrier et al., 2008). The half-size AtABCG25/WBC26 transporter, which is expressed mainly in vascular tissues, is an ABA Efflux carrier (see **Figure 1**). Overexpression of this transporter reduces water loss from leaves by facilitating the delivery of ABA to guard cells (Kuromori et al., 2010). By contrast, the full-size AtABCG40 transporter, which is primarily found in guard cells, imports ABA into stomatal cells (see **Figure 1**). This transporter is necessary for the proper plant response to ABA. Loss-of-function *abcg40* mutants have guard cells with reduced sensitivity to ABA and are more susceptible to drought stress (Kang et al., 2010). Kuromori et al. (2011) reported on a half-size ABCG transporter (AtABCG22/WBC23) that likely enhances ABA influx into guard cells (see **Figure 1**). A mutation in the gene encoding this protein results in an increase in water transpiration and drought susceptibility. However, the ABA transport activity of the corresponding protein remains to be examined.

The role of ABC transporters in ABA transport remains to be functionally analyzed. These multifaceted proteins must be fit into a broader context of functional plasticity. It is tempting to question whether a given ABC transporter or a closely related group of paralogs, which are widely expressed in various organs (e.g., leaves and roots) and/or have defined substrate specificity (ABA), participates in a coordinated manner in response to drought.

#### Secondary ABA transporters

Using a modified yeast two-hybrid system, Kanno et al. (2012) identified a nitrate transporter (NRT1.2) belonging to the NRT1/PTR (Nitrate transporter1/Peptide transporter) family currently known as ABA-IMPORTING TRANSPORTER (AIT1; see **Figure 1**). The activity of the AIT1 promoter was observed in the vascular tissues of the inflorescence stems, leaves, and roots. After the characterization of loss-of-function mutants and transport assays, it was suggested that AIT1 is an ABA importer that is essential for the regulation of stomatal aperture in the inflorescence stems. Recently, Zhang et al. (2014) identified a new ABA transporter in *A. thaliana* known as DTX50 (see **Figure 1**). This protein belongs to the DTX/MATE (The Detoxification Efflux Carriers/Multidrug and Toxic Compound Extrusion) family. Following ABA treatment, *AtDTX50* transcripts accumulated in both guard cells and the vascular bundles of leaves. Proteins encoded by *AtDTX50* localized to the PM. Transport experiments using *Escherichia coli* and *Xenopus* oocytes expressing *AtDTX50* revealed that it is an ABA exporter. AtDTX50 transport activity was the highest at an apoplastic pH of 7.0, which reflects drought conditions. Disruption of AtDTX50 activity resulted in slower wilting; this is a direct consequence of the inability to remove excess ABA and the hyperaccumulation of the hormone in leaves. In *A. thaliana* with dysfunctional DTX50, stomata closed more rapidly; this resulted in an increased tolerance to drought. Insertion mutants displayed growth retardation and ABA hypersensitivity. Overall, these results provided strong evidence that AtDTX50 plays an essential role in the control of ABA accumulation in various cell types; this protein is the second ABA Efflux transporter identified, after AtABCG25 (Zhang et al., 2014).

### ABA CONJUGATE TRANSPORTERS

It has been proposed that ABA-GE conjugates are actively transported into the vacuoles of mesophyll cells by ABC transporters (see **Figure 1**) or proton gradient-driven transporters (Burla et al., 2013). Two members of the *A. thaliana* ABCC/MRP (multidrug resistance-associated protein) subfamily, AtABCC1/MRP1 and AtABCC2/MRP2, are localized in tonoplast and exhibit ABA-GE transport activity in a yeast heterologous expression system. According to microarray analysis, the expression of ABCC2 is induced upon drought. In addition to ABCCs, secondary transporters also participate in the vacuolar sequestration of ABA conjugates; these transporters are presumably members of the MATE family (Burla et al., 2013).

### ION MOVEMENT AND STOMATAL CLOSURE UPON DROUGHT

The reliable and fast adjustment of stomatal aperture is necessary for effective drought avoidance in plants. A rapid response reduces the amount of water lost, increases water use efficiency, and allows plants to survive (Farooq et al., 2009). There are two pathways leading to stomatal closure, passive, and active. In the passive pathway, turgor loss occurs without a reduction of the solute content of the guard cells (McAdam and Brodribb, 2014). Because the passive pathway cannot sufficiently protect the plant against drought, active solute loss is necessary. The movement of osmoregulatory ions (K^+^, Cl^-^, and malate^2-^) across guard cell membranes and the gluconeogenic conversion of malate into starch, accompanied by water export through aquaporins (AQPs), results in a decrease in cell volume. Turgor is lost, and the stomata finally close. Mature guard cells have no plasmodesmata; the existence of channels, transporters, and pumps in the PM are required (Lawson and Blatt, 2014).

### Ca^2+^ PERMEABLE CHANNELS

**FIGURE 2:**
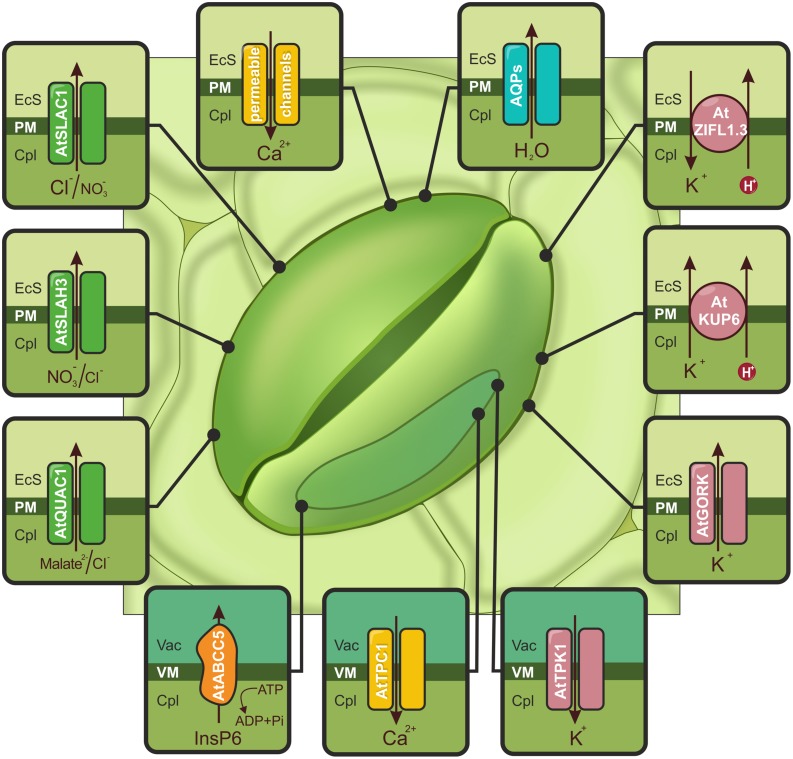
**Schematic illustration of ion channels, aquaporins (AQPs) and transporters activated by drought or ABA and controlling stomatal closure.** The accumulation of cytoplasmic Ca^2+^ ([Ca^2+^]_cyt_), is possible due to Ca^2+^-permeable channels (yellow) localized in the PM (e.g., AtCNGC5, AtCNGC6, AtGLR3.1) and the tonoplast (AtTPC1). Increasing the amount of calcium activates S-type (AtSLAC1 and AtSLAH3) and R-type (AtQUAC1) channels, which are indicated in green. AtABCC5/MRP5 (orange), localized to the vacuolar membrane (VM), is a regulator of Ca^2+^-permeable and S-type channels. The actions of the S- and R-type channels induce membrane depolarization and activate K^+^ flow through AtGORK and AtKUP6 (pink) from guard cells. The release of K^+^ from the vacuole is mediated by the AtTPK1 channel (pink). Additionally, stomatal closure is regulated by the action of the AtZIFL1.3 isoform, indicated in pink. AQPs are responsible for the outflow of water (e.g., VfPIP1), shown in blue. EcS, extracellular space; Cpl, cytoplasm; Vac, vacuole.

In the active solute loss scenario, ABA is imported into guard cells by ABCG40 in response to drought. This triggers the production of reactive oxygen species (ROS) such as hydrogen peroxide (H_2_O_2_) and nitric oxide (NO) and cytoplasmic Ca^2+^ ([Ca^2+^]_cyt_) increases (Kim et al., 2010). The level of oxidative stress is controlled by a group of low molecular antioxidant compounds known as flavonols (Watkins et al., 2014). The molecular nature of flavonol transporters in these particular cells remains to be elucidated. However, the action of such proteins indirectly influences the aperture of guard cells. Members of the MATE and ABC protein families are involved in the transport of secondary plant metabolites, including flavonoids (Zhao and Dixon, 2010). The accumulation of [Ca^2+^]_cyt_, is possible due to Ca^2+^-permeable channels located in the tonoplast and the PM (see **Figure 2**). These proteins are encoded by genes belonging to the TPC1 (two-pore channel 1), CNGC (cyclic nucleotide-gated channel), and GLR (glutamate receptor-like) families. TPC1 has been classified as slow vacuolar (SV) non-selective cation channel that is regulated by luminal Ca^2+^ (Islam et al., 2010). In *A. thaliana*, members of the CNGC and GLR families, namely AtCNGC5, AtCNGC6, and AtGLR3.1, are PM-located Ca^2+^ channels (Cho et al., 2009; Wang et al., 2013). The distinguishing feature of CNGC proteins is cGMP-dependent activation (Wang et al., 2013), which is also required for an increase in [Ca^2+^]_cyt_ (Dubovskaya et al., 2011). The long-term Ca^2+^-programmed stomatal response that prevents guard cell reopening is established by AtGLR3.1 (Cho et al., 2009).

### ANION CHANNELS (S-TYPE AND R-TYPE)

The ABA-induced signal cascade results in the activation of S-type (slow-activating sustained) and R-type (rapid-transient) anion channels, which cause PM depolarization. Simultaneously, the inhibition of H^+^-ATPase and inward K^+^ channels (KAT1/KAT2) prevents the hyperpolarization of the PM (Osakabe et al., 2014). Recent studies have identified the genes encoding S and R-type anion channels. The *A. thaliana* SLAC1 (slow anion channel-associated 1) protein is a PM-localized (Negi et al., 2008; Vahisalu et al., 2008) S-type anion channel with a preference for Cl^-^ and NO_3_^-^ (see **Figure 2**; Geiger et al., 2009; Lee et al., 2009b). This protein operates mainly in response to ABA, CO_2_, Ca^2+^, NO, and H_2_O_2_ (Vahisalu et al., 2008). Mutations in SLAC1 result in the excessive accumulation of anions, such as Cl^-^, in cell protoplasts and the disruption of stomatal closure (Negi et al., 2008; Vahisalu et al., 2008). Phosphorylation at distinct sites enables SLAC1 activation by both calcium-dependent and calcium-independent pathways downstream of ABA (Maierhofer et al., 2014). A second S-type anion channel, SLAH3 (SLAC homolog 3), is expressed in *A. thaliana* guard cells (Geiger et al., 2011). In contrast to its homolog, SLAH3 has higher permeability toward NO_3_^-^ than Cl_2_^-^ (see **Figure 2**; Geiger et al., 2009, 2011; Lee et al., 2009b). AtMRP5/ABCC5, a vacuolar inositol hexakisphosphate (InsP6) transporter from the ABC transporter family, may be a central regulator of Ca^2+^-permeable and S-type anion channels in the PM of guard cells. This regulation is possible due to the complexing of divalent cations by InsP6 or the triggering a continuous Efflux of Ca^2+^ into the cytosol by an InsP6-regulated channel (see **Figure 2**; Nagy et al., 2009). A member of the aluminum-activated malate transporter family in *A. thaliana*, QUAC1/ALMT12 (quick anion channel 1/aluminum-activated anion channel 12), has been identified and classified as malate-sensitive R-type anion channel (see **Figure 2**; Meyer et al., 2010) or a chloride and nitrate current facilitator (Sasaki et al., 2010). Loss of QUAC1 function results in impairment of stimulus-induced stomatal closure (owing to factors such as ABA, CO_2_, Ca^2+^; Meyer et al., 2010; Sasaki et al., 2010). Compared to our understanding of anion influx across the tonoplast, the molecular mechanisms of Efflux from the vacuole are poorly understood.

### POTASSIUM CHANNELS/TRANSPORTERS

Plasma membrane depolarization activates the Efflux of potassium. This transport is facilitated by GORK (guard cell outward rectifying K^+^) channels, which belong to the Shaker family of voltage-gated ion channels (see **Figure 2**). GORK transcripts have been widely observed in *A. thaliana*, especially in guard cells (Becker et al., 2003; Hosy et al., 2003), where they represent the only outward-rectifying K^+^ channel (Eisenach et al., 2014). The inhibition of GORK activity in guard cells results in defects in K^+^ Efflux and a lower rate of stomatal closure (Hosy et al., 2003). Osakabe et al. (2013) identified stress-responsive K^+^ uptake permeases (KUPs), which share redundant functions with GORK channels (see **Figure 2**). The KUP6 and KUP8 proteins in *A. thaliana* function as K^+^/H^+^ symporters. KUP6, which is highly up-regulated in response to the ABA treatment and water deficit, is localized to the PMs of guard cells, root tip cells and vascular tissues. KUP6-overexpressing lines were more tolerant to drought. A *kup68g* double mutant line (with combined *kup6* and *kup8* mutations in addition to mutation in the *gork* potassium channel) had impaired stomatal closure and ABA sensitivity; this mutant also had greater stomatal conductance and water loss rates (Osakabe et al., 2013). An isoform of the ZIFL1 (zinc-induced facilitator-like1) transporter, which belongs to the major facilitator superfamily (MFS), proved to be a modulator of stomatal aperture and conferred drought stress tolerance. The PM-localized AtZIFL1.3 protein possesses H^+^-coupled K^+^ transport activity, and its overexpression resulted in more efficient stomatal closure (see **Figure 2**; Remy et al., 2013). The main cellular depository of ions, including potassium, is the vacuole. The release of K^+^ from the vacuole is mediated by channels and transporters localized to the tonoplast. TPK1, a member of the two pore K^+^ channel (TPK) family, is responsible for vacuolar K^+^ translocation in guard cells (see **Figure 2**). The removal of TPK1 was associated with a weak phenotype; most often the stomatal closure was slower, but the opening kinetics were normal (Gobert et al., 2007).

### WATER CHANNELS

The outflow of water results in a reduction in guard cell turgor and stomatal closure; this is mediated by AQPs, which are membrane channels that belong to the MIP (major intrinsic protein) family (see **Figure 2**; Heinen et al., 2009). AQPs may form homo- and heterotetramers, with each monomer defining a single pore. Based on amino acid similarity and membrane localization, AQPs are divided into five subgroups; for a review, see (Maurel et al., 2008; Chaumont and Tyerman, 2014). The expression of several genes encoding AQPs belonging to the PIP (PM intrinsic proteins) and TIP (tonoplast intrinsic proteins) subgroups was up-regulated in leaves, mainly in the guard cells, in drought stress conditions (Heinen et al., 2009). Heinen et al. (2014) have identified *Zea mays* PIPs that are localized to stomatal complexes (mainly ZmPIP1;1, ZmPIP1;3, ZmPIP2;2) and presumably participate in stomatal movement (Heinen et al., 2014). The expression of PIP1 from *Vicia faba* and *Brassica juncea* improved drought resistance in *A. thaliana* and *Nicotiana tabacum* plants, respectively, through the promotion of stomatal closure (Cui et al., 2008; Zhang et al., 2008).

### CHANNELS/TRANSPORTERS INFLUENCING STOMATAL OPENING

Proteins that regulate stomatal movements by supporting swelling belong to a separate group of transporters. The disruption of Cl^-^ (AtCLCc, AtALMT9), K^+^ (AtNHX1, AtNHX2, AtCHX20), and NO_3_^-^ (AtNRT1.1, AtCLCa) channels/transporters, as well as the AtMRP4/AtABCC4 protein, results in a delay in stomatal opening (Guo et al., 2003; Klein et al., 2004; Padmanaban et al., 2007; Jossier et al., 2010; De Angeli et al., 2013; Andres et al., 2014; Wege et al., 2014). Phosphorylation-dependent changes in the activity of AtCLCa enable the protein to perform dual roles, in anion sequestration in the vacuole during stomatal opening and in anion release during stomatal closure, in response to ABA (Wege et al., 2014). Mutations in a malate importer protein (AtABCB14) result in accelerated stomatal closure (Lee et al., 2008). Mutations in AtALMT9, AtNRT1.1, and AtMRP4 transporters result in the impairment of stomatal responses and are associated with increased drought tolerance (Guo et al., 2003; Klein et al., 2004; De Angeli et al., 2013).

## CUTICLE DEPOSITION AS A FACET OF DROUGHT AVOIDANCE

The formation of the plant cuticle, which covers the surface of all aerial organs, is a crucial plant adaptation to terrestrial habitats. This specialized cell wall (CW) component, which is synthesized exclusively by aerial epidermal cells, consists of wax embedded in (intracuticular) and layered on (epicuticular) a lipid polyester matrix called cutin. The major function of the cuticle is to protect the plant from unfavorable environmental conditions, including drought, by limiting non-stomatal water transpiration (Yeats and Rose, 2013). In *A. thaliana*, the biosynthesis of cuticle components is induced by water deficiency (Kosma et al., 2009). Recent studies demonstrated that the increased accumulation of cuticular waxes is associated with drought resistance (Yang et al., 2011; Zhu et al., 2014) and ABA treatment (Seo et al., 2011).

During cuticle deposition, the trafficking of wax and cutin precursors occurs from the ER across the PM and CW to the plant surface (Yeats and Rose, 2013).

### INTRACELLULAR TRANSLOCATION OF CUTICULAR COMPONENTS

**FIGURE 3:**
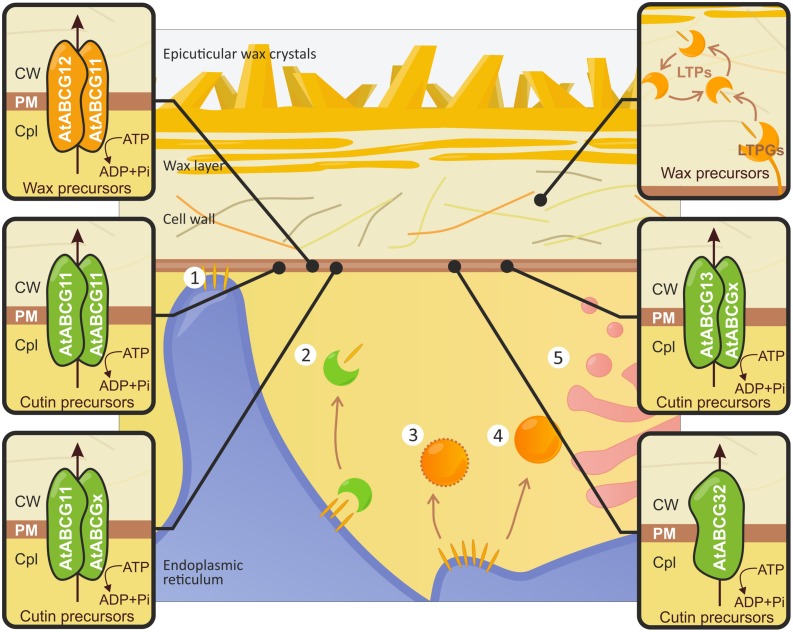
**Schematic illustration of cuticular lipid translocation.** Different scenarios describing the transport of wax precursors (orange disks) from the endoplasmic reticulum (ER) to the PM have been proposed. Cuticular lipids may be (1) relocated at ER-PM contact sites, (2) grasped by ACBPs (e.g., AtACBP1; green crescent-shaped bodies), transported by (3) coated oleophilic bodies, (4) uncoated vesicles, or (5) the Golgi-mediated secretory pathway. The translocation of wax and cutin precursors through the PM is a complex action involving several ABC transporters, which are indicated in orange and green, respectively. The latter are either half-size proteins (AtABCG11, AtABCG12, AtABCG13), which function as homo- or heterodimers, or full-size transporters (AtABCG32). Often composition of the homo- or heterodimers defines the profile of the transported substrate (see the text for details). The transport of wax precursors across the cell wall to the plant surface is mediated by LTPGs (e.g., AtLTPG1 and AtLTPG2) in association with yet unknown LTPs. CW, cell wall; Cpl, cytoplasm.

To date, the intracellular mechanism of wax and cutin precursor trafficking remains unclear. Several hypothetical pathways to the PM have been suggested (see **Figure 3**). These precursors might reach the PM via the following routes: (i) direct relocation at ER-PM contact sites (Samuels and McFarlane, 2012); (ii) transport by cytosolic carrier proteins (e.g., Acyl-CoA binding proteins – ACBPs; Li-Beisson et al., 2010); (iii) vesicular translocation in oleosome bodies coated by oleosin-like proteins; (iv) translocation in uncoated vesicles that sequestrate the cuticular lipids into lipid rafts (Schulz and Frommer, 2004); or (v) Golgi mediated exocytosis (Samuels et al., 2008). Through mutant analysis combined with live cell imaging, electron microscopy and chemical phenotyping, McFarlane et al. (2014) demonstrated that the movement of intracellular wax requires vesicle traffic mediated by Golgi- and *trans*-Golgi networks. However, AtACBP1 has been shown to contribute to the formation of the stem cuticle; dysfunction in this protein resulted in a reduction in wax and cutin monomer composition. Moreover, AtACBP1 exhibits biding activity to wax precursors (Xue et al., 2014). The cytosol-localized protein LjLTP3, a member of the LTP (lipid transfer protein) family, is able to bind lipids and confers drought tolerance in *Lotus japonicus*.

### TRANSPORT OF CUTIN AND WAX PRECURSORS ACROSS THE PLASMA MEMBRANE

Members of the ABC transporter family mediate the flux of cuticular components through the PM. Two half-size ABC transporters, named AtABCG12/WBC12/CER5 (Pighin et al., 2004) and AtABCG11/WBC11/DSO/COF1 (Bird et al., 2007; Luo et al., 2007; Panikashvili et al., 2007; Ukitsu et al., 2007), are involved in the secretion of cuticle precursors into the apoplastic environment (see **Figure 3**). Mutation of both genes results in a significant reduction in the stem wax load (approx. 50%); a major depletion in C29 alkane, the major component of cuticular waxes in stems, is also observed (Pighin et al., 2004; Bird et al., 2007). The total amount of wax in the epidermis remains unchanged, indicating that the absence of these two proteins impedes wax transport rather than biosynthesis. AtABCG11 is also likely involved in the translocation of cutin monomers in vegetative and reproductive organs and the transport of suberin in roots (Panikashvili et al., 2007, 2010). The evidence that AtABCG11 forms homo- and heterodimers with AtABCG12 comes from bimolecular fluorescence complementation and protein trafficking assays (McFarlane et al., 2010). AtABCG11 also plays a dominant role in the created complex; ABCG12 could not traffic to the PM in the absence of ABCG11, whereas ABCG11 trafficking was independent of ABCG12 (McFarlane et al., 2010). The half-size ABCG transporter from *Solanum tuberosum* (StABCG1) was shown to contribute to the export of suberin components across the PM (Landgraf et al., 2014). Silencing of the *StABCG1* gene, which is expressed mainly in the roots and tuber periderm, results in a reduction in esterified suberin monomers; this is accompanied by an accumulation of the presumed suberin precursors. Root and tuber morphology was altered, and tubers were more susceptible to drought (Landgraf et al., 2014). The putative ortholog of AtABCG12 found in the moss species *Physcomitrella patens* is involved in the transport of a wax precursor. This finding suggests that the role of ABCG transporters, particularly half-size transporters, in the movement of cuticle components is evolutionarily conserved among land plants (Buda et al., 2013). Loss-of-function in *AtABCG13/WBC13* (another half-size ABCG transporter that belongs to the ABCG12 clade) results in a significant depletion in petal cutin monomers; this result indicates that the protein plays a role in cutin deposition in flowers (see **Figure 3**; Panikashvili et al., 2011). The full-size ABCG transporter (AtABCG32) has been shown to contribute to the formation of functional cuticles (see **Figure 3**; Bessire et al., 2011). Knockout mutations in AtABCG32 result in a significant increase in cuticle permeability in leaves and flowers, as well as in water loss from rosettes and herbicide sensitivity. The ultrastructural changes in the cuticle were associated with reduced amounts of minor aliphatic cutin monomers; these monomers are most likely exported from epidermal cells by AtABCG32. The AtABCG32 monocot orthologs OsABCG31 1 and HvABCG31 may also be involved in cuticle deposition. Loss-of-function mutations in these genes, both in *Oryza sativa* and in *Hordeum vulgare*, result in water retention deficiency phenotypes (Chen et al., 2011).

Members of the ABCG subfamily in *A. thaliana* are also required for proper pollen coat (extracellular matrix) and exine (outer wall) formation. These structures protect gametophytes in unfavorable environmental conditions. Dysfunction in AtABCG9/WBC9 and AtABCG31 proteins results in reduced steryl glycoside levels and altered pollen coat morphology. Correct morphology is essential for pollen coat maturation. A double mutant exhibited increased water loss upon exposure to dry air (Choi et al., 2014). The AtABCG26/WBC27 protein participates in exine deposition in the pollen, presumably by transporting sporopollenin precursors (Quilichini et al., 2010; Choi et al., 2011; Dou et al., 2011). An ortholog in *O. sativa* (OsABCG15) is crucial for anther cuticle and pollen exine formation (Wu et al., 2014).

### TRANSPORT OF CUTICULAR COMPONENTS THROUGH THE CELL WALL TO THE CUTICLE

Once cuticular components have been moved across the cell membrane, they must pass the hydrophilic CW to reach the cuticle. Movement through the extracellular matrix appears to be facilitated by LTPs. Two members of the LTP family (LTPG1 and LTPG2) are defined as GPI-anchored (glycosylphosphatidylinositol –anchored) proteins. These proteins are located at the exterior face of the PM and are possibly involved in wax export (see **Figure 3**; Debono et al., 2009; Lee et al., 2009a; Kim et al., 2012). Disruption of AtLTPG1 or its homolog AtLTPG2 modifies the structure of the cuticular layer and reduces the amount of C29 alkane; this is also seen in AtABCG12 and AtABCG11 mutants (Debono et al., 2009; Lee et al., 2009a; Kim et al., 2012). The expression of LTPG1 can complement wax scarcities in *ltpg1* mutant lines (Debono et al., 2009). Compared to each single mutant, the double mutant exhibited a more severe phenotype (Kim et al., 2012). Taken together, it seems likely that LTPG1 and LTPG2 are functionally overlapping wax exporters. These proteins act either directly by associating with ABC transporters or indirectly by creating suitable environment for wax Efflux (Debono et al., 2009; Kim et al., 2012). The former model assumes that LTPGs are loaded with C29 alkane cargo and transfer it to other extracellular LTPs, which then carry the lipid load to the plant surface (Debono et al., 2009).

## ROOT RESPONSES UPON DROUGHT

In addition to mechanisms that reduce water loss in the aerial parts of plant, drought avoidance also requires strategies based on the regulation of root water uptake; this is achieved by modulating root growth and hydraulic conductivity (Aroca and Ruiz-Lozano, 2012).

### TRANSPORTERS PARTICIPATING IN ROOT GROWTH AND DEVELOPMENT

To increase the volume of potentially accessible water, the integrated action of ABA and auxins promotes root growth and development (Xu et al., 2013b). Several transporters, which belong to distinct gene families, act as key modulators of auxin translocation and subsequent root morphology changes (Petrasek and Friml, 2009; Zazimalova et al., 2010). There are only few examples of extensive crosstalk between the mechanisms that regulate auxin transport, root development, and drought resistance. The expression of an *A. thaliana* vacuolar H^+^-pyrophosphatase (AVP1) confers tolerance against drought in many plant species, including *Lycopersicon esculentum* (Park et al., 2005), *Saccharum officinarum* (Kumar et al., 2014b), *N. tabacum* (Arif et al., 2013), and *Gossypium hirsutum* (Pasapula et al., 2011). AVP1 overexpressing plants exhibited greater PP_i_ -driven sequestration of ions and sugars into the vacuole, increased water retention and increased cell turgor. Auxin transport is also stimulated, enhancing root system development (Park et al., 2005). This adaptation to stress conditions is also possible due to the different subcellular and tissue distribution of isoforms of transporters with similar substrate profiles. The MFS carrier ZIFL1 protein (Remy et al., 2013) is one example. The two splice isoforms of ZIFL1, ZIFL1.1, and 1.3, both have K^+^ transport activity that likely influences membrane proton gradients, resulting in distinct biological roles in roots and guard cells, respectively. The change of pH that occurs as a consequence of K^+^ translocation results either in stomata closure or the modulation of auxin transport. In this way, plants can control stomata aperture and change root morphology if necessary.

The effects of ABA on root morphogenesis differ between various plant species, especially the effects on the formation of the lateral roots. In *A. thaliana*, ABA inhibits lateral root formation. In legumes such as *Medicago truncatula*, ABA promotes the growth of lateral roots and affects the nodulation process. ABA also acts as a negative regulator of the early stages of nodulation (affecting epidermal Nod factor signaling and cytokinin-activated cortex cell division) and suppresses symbiotic nitrogen fixation (Liang et al., 2007; Ding and Oldroyd, 2009). Nodule formation is an energetic cost that must be strictly controlled; nodulation is highly influenced by environmental conditions. It has been proposed that alternations in hormone levels, especially stress hormones such as ABA, serve as shoot derived signals directed at the roots (Ding and Oldroyd, 2009). This fluctuation in ABA levels is the bridge joining nodulation control and the environmental status of the plant. Despite their importance, the mechanisms behind the translocation/control of such signals are still largely unknown.

### CHANGING ROOT HYDRAULIC CONDUCTIVITY – AQUAPORINS TAKE ACTION

Root hydraulic conductivity (L), the water flux across the root surface area, is governed by drought and ABA. In water deficit conditions, root hydraulic conductivity decreases to prevent water outflow from the roots due to progressively decreasing soil water potential (Aroca et al., 2012). Morphological changes in the root tissue and the existence of water channels constitute the main long-term and short-term determinants of root hydraulic conductivity, respectively. The formation of apoplastic barriers, such as Casparian bands and suberin lamellae, prevents cellular death by limiting water outflow into the soil (Lobet et al., 2014). The half-size ABCGs, StABCG1, and AtABCG11, affect the root suberin content. Therefore, these proteins might be responsible for the proper formation of suberin lamellae (Panikashvili et al., 2010; Landgraf et al., 2014). Rapid and reversible alterations in water permeability are done by controlling the expression and activity of AQPs. AQPs allow for the passive movement of water through a cell-to-cell pathway, which is the major water path during drought (Aroca and Ruiz-Lozano, 2012). The expression of the two aforementioned MIP subgroups, PIPs and TIPs, is greater in roots than in leaves. Moreover, the localization of PIPs has been associated with the presence of root hydrophobic barriers; this finding suggests that these proteins are involved in the regulation of water movement during drought (Chaumont and Tyerman, 2014). Upon water deficit, the expression of several PIPs is down-regulated to reduce water loss (Moshelion et al., 2014). According to Jang et al. (2004), nine out of the thirteen PIP genes expressed in the root (three from the PIP1 group and six from PIP2 group) showed significant reductions in mRNA accumulation in *A. thaliana* plants exposed to drought. Similarly, in *N. tabacum*, the expression of PIPs is significantly down-regulated in a manner corresponding to the level of drought stress (Mahdieh et al., 2008); this phenomenon has also been observed in *Fragaria vesca* (Surbanovski et al., 2013) and *Camellia sinensis* roots (Yue et al., 2014). Additionally, the ectopic overexpression of *A. thaliana* PIP1;4 and PIP2;5 in *N. tabacum* resulted in increased water loss under dehydration stress (Jang et al., 2007). By contrast, the heterologous expression of the *Triticum aestivum* aquaporin gene (TaAQP7), which belongs to PIP2 subgroup, conferred drought tolerance in transgenic *N. tabacum*; plants were able to maintain their water status, reduce the accumulation of ROS and prevent membrane damage via an ABA-dependent pathway (Zhou et al., 2012). Concerning TIP AQPs, plants may respond to drought through cell elongation, osmotic adjustment, and the mediation of water transport across the tonoplast (Wudick et al., 2009). The constitutive expression of TIP1 from *Panax ginseng*, TIP1;2 from the highly drought-resistant *Thellungiella salsuginea* and TIP2 from *Solanum lycopersicum* confer tolerance to water deprivation in *A. thaliana*; an increase in water absorption requires roots elongation and high water permeability in the tonoplast (Peng et al., 2007; Sade et al., 2009; Wang et al., 2014). Conversely, it has been demonstrated that TIPs from *Glycine soja* (TIP2;1) and *T. aestivum* (TIP2;2) have a negative impact on *A. thaliana* drought tolerance (Wang et al., 2011; Xu et al., 2013a).

### ARBUSCULAR MYCORRHIZA IMPROVES WATER UPTAKE CAPACITY

Increasing evidence indicates that arbuscular mycorrhiza (AM) play a crucial role in enhancing tolerance to drought (Abbaspour et al., 2012; Li et al., 2013, 2014; Barzana et al., 2014). Glomeromycota fungi are intimately associated with 80% of land plants; these organisms, supply water and nutrients to the roots of host plants in return for carbon compounds (Schmitz and Harrison, 2014). The improvement in water status is possible by harnessing the hyphae growing in the soil, enlarging the absorption surface, and exploiting the ability of the fungus to take up water despite the low soil water potential (Ruiz-Lozano et al., 2012). In addition, AM symbiosis affects the hydraulic properties of roots and maintains root water permeability by the regulating the expression of plant AQPs (PIPs and TIPs) and the activity of fungal AQPs (Li et al., 2013; Barzana et al., 2014). Considering the involvement of AM in plant response to drought stress, transport mechanisms play a crucial role in the establishment of plant–fungus interactions. The successful recognition of root-exuded plant metabolites, in particular flavonoids (F), 2-hydroxy fatty acids (2OH-FA), strigolactones (SL), and 16C cutin monomers, is a prerequisite for host-root colonization via hyphopodium development (Nadal and Paszkowski, 2013). Despite the importance of these transporters, the knowledge of this pre-symbiotic molecular crosstalk is limited. The isolation of the *Petunia hybrida* PDR1 transporter has shed light on SL transport, as it encodes for a putative root SL exporter (Kretzschmar et al., 2012). A corresponding protein has been found in the PM of hypodermal passage cells (HPC), the non-suberized cortical entry points for AM hyphae (Kretzschmar et al., 2012). The entrance into cortical cells, where hyphae develop a treelike structure, is called an arbuscule. Arbuscules form through the PM and tonoplast invagination. Formed arbuscules are enveloped by a plant-derived periarbuscular membrane (PAM), a key symbiosis interface that contains several plant transporters and mediates bi-directional nutrient flux (Recorbet et al., 2013).

## OSMOTIC ADJUSTMENT AT THE CELLULAR LEVEL AS A FACET OF DROUGHT TOLERANCE

Most plants have the capacity to tolerate low water content by increasing cellular osmolarity, thereby protecting cells from damage due to dehydration. This acclimating process involves the accumulation of an array of highly concentrated, osmotically active compounds in the cytosol, rather than in the vacuole, without the interference of primary metabolism. Osmotic regulators include inorganic ions and compatible solutes, such as sugars, sugar alcohols (polyols and cyclitols), amino acids [particularly proline (Pro)], and quaternary ammonium compounds (QACs), notably glycine betaine (GB; Sanders and Arndt, 2012). ABA participates in the accumulation of the aforementioned protectants. Therefore, ABA serves as a bridge between avoidance and tolerance responses. Strategies for achieving high levels of osmoregulatory solutes to cope with water deprivation are based either on altering metabolism or transport. Early studies indicated that the movement and differential distribution of these compounds, both within and between cells, is regulated by membrane-associated transporters; most of these transporters are secondary active transporters.

### AMINO ACIDS AND QACs TRANSPORTERS

#### Proline transporters

Proline accumulation is the first response of plants subjected to a range of environmental stressors, including drought. The correlation between this widely occurring amino acid and drought tolerance has been demonstrated in several plant species (Javed et al., 2014; Kumar et al., 2014a; Ravikumar et al., 2014; Singh et al., 2014). In addition to its well-established role in osmotic adjustment, Pro contributes to stress tolerance by functioning as a molecular chaperon that stabilizes subcellular structures, as a free radical scavenger and as stress-signaling molecule. During periods of stress, Pro is synthesized in the chloroplast and cytosol and then transported. Pro is degraded within root mitochondria (Szabados and Savoure, 2010) to provide energy, which beneficial for the accumulation of root biomass (Kavi Kishor and Sreenivasulu, 2013).

**FIGURE 4:**
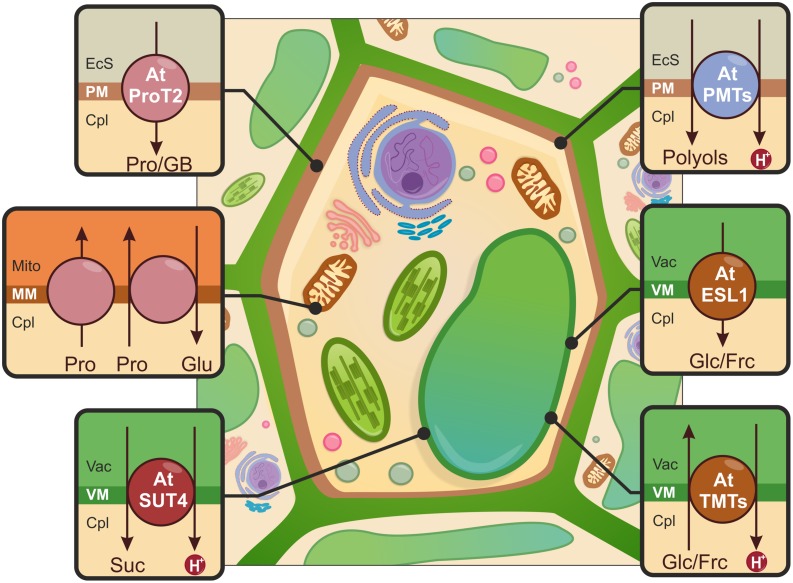
**Schematic illustration of the sub-cellular distribution of osmotically active compounds upon drought stress.** Proline (Pro) and glycine betaine (GB) transport (pink) across the PM is mediated by Pro transporters (e.g., AtProT2). Pro translocation through the mitochondrial membrane (MM) is possible due to Pro carriers that function as uniporters and Proline/Glutamate (Pro/Glu) antiporters. Glucose (Glc)/fructose (Frc) influx and Efflux across the tonoplast (VM) is driven by Tonoplast Monosaccharide Transporters (e.g., AtTMT1 and AtTMT2) and Early Responsive to Dehydration six-like 1 carrier (AtESL1), indicated in brown. Sucrose (Suc) transporters (e.g., AtSUT4, red) participate in Suc export from the vacuole. The import of polyols into the cell is possible due to polyol/monosaccharide transporters (e.g., AtPMT1, AtPMT2, AtPMT5), shown in blue. EcS, extracellular space; Cpl, cytoplasm; Mito, mitochondria; Vac, vacuole.

Transporters that recognize Pro have been classified into the following three subfamilies: (i) the amino acid permease (AAP) subfamily; (ii) the lysine–histidine transporter (LHT) subfamily; and (iii) the Pro transporter (ProT) subfamily. Members belonging to the AAP and LHT subfamilies are low selectivity proline transporters and participate in the translocation of neutral amino acids (Rentsch et al., 2007; Lehmann et al., 2010). Three ProTs have been identified in *A. thaliana* (Grallath et al., 2005). The expression profiles of the identified genes revealed that the ProT1 and ProT2 mRNA is accumulated when Pro levels are elevated. During progressive drought, only the ProT2 gene was strongly up-regulated. Grallath et al. (2005) observed that GUS expression driven by the ProT2 promoter occurred in the root epidermis and cortex. The corresponding protein has been found in the PM. AtProT2 exhibits Pro transport activity (Lehmann et al., 2011), and may facilitate Pro import across the PM under stress conditions (see **Figure 4**).

Transport mechanisms across chloroplast membranes, where Pro is synthesized, remain elusive. Two transport systems for Pro uptake into the mitochondria have been demonstrated (Di Martino et al., 2006). A study using *Triticum durum* seedlings confirmed that Pro enters the mitochondria through the uniporter (see **Figure 4**); in the mitochondria, Pro is further catabolized to glutamate (Glu). Glu is then exported from the mitochondria in exchange for Pro via the Pro/Glu antiporters (see **Figure 4**).

#### Glycine betaine transporters

Mass synthesis of GB occurs in response to water limitations and ABA treatment (Zhang et al., 2012). Comprehensive studies using transgenic plants such as *Spinacia oleracea, S. lycopersicum,* and *O. sativa* have demonstrated that GB accumulation results in improved plant tolerance to drought stress (Shirasawa et al., 2006; Zhang et al., 2011). The transport activity of GB has been demonstrated using ProTs from *A. thaliana*, *Beta vulgaris*, *H. vulgare*, *S. lycopersicum*, and *Avicennia marina* (Lehmann et al., 2011). The uptake of GB and Pro has been shown to be regulated by pH (Takabe, 2012).

### SUGAR AND SUGAR ALCOHOL TRANSPORTERS

An increased synthesis of sugar and polyol compounds is triggered by various abiotic stresses. However, types of accumulated sugars may differ among plant species and nature of abiotic stressors (Zhuo et al., 2013; Folgado et al., 2014; Moyankova et al., 2014). Within the cell, carbohydrates are stored in chloroplasts and in the vacuole in the forms of starch and soluble sugars, respectively. In unfavorable conditions, the cytosolic concentrations of sugars and their derivatives are regulated by membrane-embedded transporters. There are three major families of mono- and disaccharide transporters, as follows: monosaccharide/polyol transporters (MST), sucrose transporters/carriers (SUT/SUC) and the SWEET sugar family of transporters (Lalonde and Frommer, 2012). The vast majority of sugar transport is an active process, requiring a proton motive force. However, recent findings indicate that sugar-specific facilitated diffusion transporters also exist in plant cells.

#### Monosaccharide transporters

Two tonoplast-localized, secondary transporters from *A. thaliana* are candidate vacuolar hexose loading proteins (see **Figure 4**). These transporters are TMT1 and TMT2 (Tonoplast Monosaccharide Transporters), which belong to the MST family. The expression of these proteins was up-regulated upon drought treatment. Mutation in the TMTs resulted in the decreased accumulation of glucose and fructose. The direct involvement of AtTMTs in energy-dependent vacuolar monosaccharide transport has been demonstrated in a glucose uptake assay using leaf mesophyll vacuoles isolated from wild-type and knockout lines (Wormit et al., 2006).

While the influx of hexoses into the vacuole is thermodynamically active, Efflux is assumed to be a passive process. Efflux is mediated by members of the early responsive to dehydration 6-like (ERD6) protein group, one of the clades within the MST family. Three members have been characterized thus far; only two have been described in the context of drought stress (Yamada et al., 2010; Poschet et al., 2011). The first identified gene, *ERD6*, was isolated from a cDNA library obtained from *A. thaliana* plants subjected to water deprivation (Kiyosue et al., 1998). Further phylogenetic analysis of the ERD6-like family revealed a close homologue of ERD6, the tonoplast-localized ESL1 (ERD six-like 1; see **Figure 4**; Yamada et al., 2010). The accumulation of *AtESL1* mRNA was observed after drought and the exogenous application of ABA. GUS staining was detected mainly in pericycle and xylem parenchyma cells of roots. To obtain direct evidence that AtESL1 is a functional monosaccharide transporter, a heterologous expression system in BY-2 cells was used. Translocation ability was not altered by a reduction in the proton gradient, indicating that ESL1 acts as a facilitated diffusion carrier (Yamada et al., 2010).

#### Disaccharide transporters

Our understanding of the involvement of SUT in drought tolerance is limited. Among several described SUTs, only one transporter from *P. tremula* (PtaSUT4), one from *O. sativa* (OsSUT2) and two from *A. thaliana* (AtSUC2 and AtSUC4), may be relevant in a drought tolerance context; these proteins are tonoplast symporters. *PtaSUT4* is predominantly present in the tonoplast of mesophyll cells in stems and source leaves (Payyavula et al., 2011). RNAi-mediated silencing of *PtaSUT4* resulted in the increased vacuolar sequestration of SUC; this promoted leaf growth and reduced of water uptake and movement in the xylem. Simultaneously, concentration of raffinose family oligosaccharides (RFOs) has also been reduced, implying the role of PtaSUT4 in regulation of osmotic gradients between cellular compartments in response to water stress (Frost et al., 2012). RFOs serve as compatible solutes for protection against abiotic stresses especially drought (Taji et al., 2002), and are synthesized in the cytosol mainly from sucrose (den Ende, 2013). Therefore, suppression of *PtaSUT4* caused a decline in cytosolic sucrose pool necessary for RFOs biosynthesis upon stress conditions. The SUT protein from *O. sativa* (OsSUT2) is a tonoplast-localized H^+^-Suc symporter (Eom et al., 2011). GUS activity was detectable in all of the examined *O. sativa* tissues, the highest *OsSUT2* expression was observed in leaf mesophyll cells. In an analysis of primary carbon metabolites, an increased accumulation of Suc in the leaves of *ossut2* mutant plants was observed; this might be the result of decreased sugar export activity in the vacuole. Changes in *OsSUT2* expression upon drought treatment have not been observed (Eom et al., 2011). In a parallel study, Ibraheem et al. (2011) demonstrated the strong up-regulation of *OsSUT2* during water stress. Two *A. thaliana* SUT proteins involved in the drought stress response have been identified (Gong et al., 2014). The loss-of-function mutant lines for AtSUC2 and AtSUC4 displayed hypersensitivity to drought and ABA treatment; this was due to changes in sucrose distribution in the shoots and roots and to sucrose accumulation in source organs. In response to drought and ABA treatment, *AtSUT4* and *AtSUT2* expression was induced in the minor veins of leaves where high-capacity sucrose transport is needed for phloem loading (Gong et al., 2014). The heterologous expression of AtSUC4 in yeast confirmed Suc transport ability (see **Figure 4**; Weise et al., 2000). Overall, the results suggest a putative role for AtSUC2 and AtSUC4 in Suc flux under drought stress conditions.

#### Sugar alcohol transporters

Six genes belonging to the polyol/monosaccharide transporter (PMT) subfamily have been identified in *A. thaliana*; these belong to the MST family. However, only AtPMT5, AtPMT1, and AtPMT2 have been partially characterized (see **Figure 4**; Klepek et al., 2005, 2010; Reinders et al., 2005). Simultaneous analyses of AtPMT5 described this protein as a broad-spectrum H^+^ symporter for polyols, cyclitols and numerous monosaccharides that resides in the PM (Klepek et al., 2005; Reinders et al., 2005). Considering its wide substrate specificity, a ubiquitous expression pattern and an unchanged mutant phenotype under testing conditions (e.g., drought), the physiological functions of AtPMT5 remain unclear and require further investigation (Klepek et al., 2005). AtPMT1 and AtPMT2 catalyze the energy-dependent PM transport of xylitol and fructose in pollen grains and young xylem; these proteins may also play a role in plant CW modifications (Klepek et al., 2010).

## CONCLUSION

It is predicted that water deficits will continue to be a major abiotic factor affecting global crop yields. Genetic engineering for drought resistance using reported candidate genes in major food crops is in progress.

Recent discoveries in plant transporters, e.g., the coordinated action of ABA exporters and importers that control the stomatal pore, points directly toward candidate genes that may have innovative applications. However, the underlying message from basic research is that many fundamental mechanisms influencing drought adaptation remain to be uncovered. The existence of homologs from multigenic families (e.g., ABC proteins) and spliced isoforms (e.g., ZIFL1) illustrate that molecular transport can have various local tissue/organ effects that result in coordinated plant adaptation to drought stress. Understanding the complexity of such responses will be the main challenge in upcoming years.

The knowledge acquired must be adapted to particular evolutionary backgrounds. For legumes (the second most important family of crop plants after *Poaceae*), targeting drought tolerance via the genetic manipulation of a particular organ (e.g., root) during especially susceptible stages such as nodulation could be important. However, the legume-specific reactions of roots to ABA, combined with nodulation control and environmental status, force researchers to propose new legume-specific approaches. It is expected that the research into the fundamental mechanisms of plant membrane transport processes will continue. New crop varieties will be produced, and new avenues toward more sustainable and productive agriculture will open up, in spite of the impending challenges.

## Conflict of Interest Statement

The authors declare that the research was conducted in the absence of any commercial or financial relationships that could be construed as a potential conflict of interest.
